# Enhancing succinic acid biosynthesis in *Escherichia coli* by engineering its global transcription factor, catabolite repressor/activator (Cra)

**DOI:** 10.1038/srep36526

**Published:** 2016-11-04

**Authors:** Li-Wen Zhu, Shi-Tao Xia, Li-Na Wei, Hong-Mei Li, Zhan-Peng Yuan, Ya-Jie Tang

**Affiliations:** 1 School of Public Health, Wuhan University, Wuhan 430071 China; 2 Key Laboratory of Fermentation Engineering (Ministry of Education), Hubei Key Laboratory of Industrial Microbiology, Hubei Provincial Cooperative Innovation Center of Industrial Fermentation, Hubei University of Technology, Wuhan 430068 China

## Abstract

This study was initiated to improve *E. coli* succinate production by engineering the *E. coli* global transcription factor, Cra (catabolite repressor/activator). Random mutagenesis libraries were generated through error-prone PCR of *cra*. After re-screening and mutation site integration, the best mutant strain was Tang1541, which provided a final succinate concentration of 79.8 ± 3.1 g/L: i.e., 22.8% greater than that obtained using an empty vector control. The genes and enzymes involved in phosphoenolpyruvate (PEP) carboxylation and the glyoxylate pathway were activated, either directly or indirectly, through the mutation of Cra. The parameters for interaction of Cra and DNA indicated that the Cra mutant was bound to *aceBAK*, thereby activating the genes involved in glyoxylate pathway and further improving succinate production even in the presence of its effector fructose-1,6-bisphosphate (FBP). It suggested that some of the negative effect of FBP on Cra might have been counteracted through the enhanced binding affinity of the Cra mutant for FBP or the change of Cra structure. This work provides useful information about understanding the transcriptional regulation of succinate biosynthesis.

Succinate, which is an intermediate in the tricarboxylic acid cycle (TCA), has many applications in food, agricultural, and pharmaceutical industries^1^, and this molecule has been identified as one of the 12 most valuable bulk chemicals^2^. As the end product of energy metabolism, succinate can be produced by many microbes^3^, and *Escherichia coli* has been widely engineered for succinate production^4^,^5^,^6^.

Most cellular phenotypes are controlled by several genes. However, current metabolic engineering approaches are limited by multiple gene modifications^7^. The metabolic networking has a robust effect^8^, and simple genetic manipulation is sometimes ineffective. Therefore, study of global regulation through multiple, simultaneous perturbations is necessary. Transcription factors (TFs) are important components of gene expression regulation networks and respond to changes in the cellular environment by altering the expression of relevant genes^9^. Prokaryotes have complex mechanisms to regulate gene transcription through the action of TFs^10^. In recent years, transcriptional engineering approaches have been applied to strain engineering and address the limitations of classical metabolic engineering approaches; thus, these techniques have been proven useful for improving cell phenotype^11^,^12^. However, to the best of our knowledge, no work has been done to improve succinate biosynthesis using a transcriptional engineering approach.

Cra (catabolite repressor/activator) has been shown to play a role in the global regulation of genes pertaining to carbon metabolism^13^. Cra is a dual transcriptional regulator that plays a pleiotropic role to modulate the direction of carbon flow through various energy-related metabolic pathways^14^. For this reason, Cra is implicated in the expression of a large number of operons encoding enzymes that constitute the central pathways of carbon metabolism. Cra activates genes that are involved in the Krebs cycle and the glyoxylate shunt^15^ and negatively affects genes that are related to the Entner Doudoroff^16^ and glycolytic^14^ pathways.

In this study, the global regulator Cra was first engineered using error-prone PCR. The relative expression and activity of the key genes and enzymes involved in succinate metabolism were then determined. The structure of the Cra mutant and the properties of the Cra-effector and Cra-DNA complex were also investigated to clarify the transcriptional regulation mechanism of Cra. Thus, this study provides useful information about the transcriptional regulation of succinate biosynthesis.

## Materials and Methods

### Strains, plasmids, and chemicals

All strains and plasmids used in this study are listed in Table 1. The primers used are summarized in Table 2. During strain construction, the strains were cultured aerobically at 37 °C in Luria broth (5 g/L yeast extract, 10 g/L tryptone, and 10 g/L NaCl). *E. coli* strain DH5α was used for plasmid construction. *E. coli* BL21 was used for the expression and purification of Cra, and *E. coli* AFP111 was kindly provided by Dr. David P. Clark at Southern Illinois University^17^. The plasmids pTrc99A and pET28a were used as foundation plasmids to prepare constructs and for overexpression. rTaq DNA polymerase was purchased from Takara (Takara, Dalian, China). Pfu DNA polymerase was obtained from Fermentas (Burlington, Ontario, Canada). Restriction enzymes and T4 DNA ligase were obtained from New England Biolabs (Ipswich, USA), and the cycle-pure kit, the gel extraction kit, the bacterial RNA kit, and the plasmid mini kit were obtained from Omega (Omega Bio-Tek, Doraville, USA).

### Construction of error-prone PCR libraries

Error-prone PCR was performed using a random mutagenesis kit (Fanke, Shanghai, China) according to the manufacturer’s protocols. The PCR products were purified and digested using BamHI and EcoRI, inserted into digested pTrc99A plasmid, and ligated using T4 DNA ligase. Transformation was carried out by electroporation (BTX, USA) using *E. coli* AFP111 competent cells.

### Rapid screening method

A 96-well plate assay was used to screen for mutant strains of high succinate production^18^. Mutants were spread on LB plates that contained 100 μg/mL ampicillin, 50 μg/mL kanamycin, and 10 μg/mL chloramphenicol, and then incubated at 37 °C for 12 h, after which the colonies were transferred to 96-well plates containing 1 mL of fermentation medium. After 10 h of incubation, the culture medium in each well was removed by centrifugation and replaced by fresh medium; the plates were then incubated at 37 °C for 72 h under CO_2_. The average succinic acid concentration in each column on the plate (i.e., each set of eight wells) was measured using HPLC to determine which column had the greatest average value. Then, each well in the selected column was measured to determine which mutant strain yielded the greatest concentration of succinate. The selected strains were subsequently screened in anaerobic bottles.

### Anaerobic bottle culture

The pre-culture and fermentation medium comprised the following (g/L): glucose 40, tryptone 20, yeast extract 10, K_2_HPO_4_·3H_2_O 0.9, KH_2_PO_4_ 1.14, CaCl_2_· 3H_2_O 0.25, (NH4)_2_SO_4_ 3.0, and MgSO_4_·7H_2_O 0.5. For the first pre-culture, fresh colonies were selected from LB agar plates that were supplemented with ampicillin (100 mg/L), chloramphenicol (34 mg/L), and kanamycin (34 mg/L); the colonies were then inoculated into 250-mL flasks containing 50 mL Luria broth and were grown at 37 °C and 180 rpm for 10 h. For the second pre-culture, 50 mL of the seed medium was prepared in a 250-mL rotary shaker and then inoculated with 200 μL of the first preculture broth; the culture was then incubated for 10 h at 37 °C on a rotary shaker at 180 rpm. For the fermentation, 5% (v/v) of the second pre-culture was inoculated in anaerobic bottles containing 50 mL of fermentation medium. After aerobic culture at 37 °C and 180 rpm for 4 h, 40 g/L MgCO_3_ was added, a small amount of CO_2_ was vented, and the cells were transferred to anaerobic conditions via rubber. The experiments were sampled at 72 h, and three replicates were performed in parallel.

### Fed-batch culture

The preculture medium and conditions have previously been described^4^. Dual-phase fed-batch fermentation was conducted using 5 L of initial fermentation medium in a 7.5-L Bioflo 115 fermenter (New Brunswick Scientific, USA). During aerobic fermentation, the initial sugar concentration was 35 g/L. The dissolved oxygen (DO, approximately 40%) was controlled by adjusting the agitation speed and aeration rate. After cultivation for 15 h, the residual sugar concentration was less than 1 g/L, and the aerobically grown cells were directly transferred to anaerobic conditions. The glucose concentration was adjusted to 40 g/L by supplying glucose as an 800 g/L solution. A rotation speed of 400 rpm and 0.1 vvm of external CO_2_ gas were used. The pH was maintained at 7.0 using 20 g/L MgCO_3_ and 5 M NaOH. In the subsequent fermentation, whenever the residual sugar concentration fell to less than 10 g/L, glucose was added to achieve 40 g/L. Determination of the cell mass and measurements of the residual sugar and organic acid concentrations were performed as previously described^4^.

### RT-qPCR

*E. coli* AFP111 cells transformed with plasmids were collected at the onset of anaerobic culture (15 h) during the dual-phase fed-batch fermentation, and total RNA was extracted using a bacterial RNA kit (Omega Bio-Tek, Doraville, USA). Total RNA fragments were reverse-transcribed to cDNA using the PrimeScript^TM^ RT reagent kit (Takara, Dalian, China). 16S rRNA was selected as the endogenous control. All cDNA samples were diluted to a final concentration of 10 ng/μL. A two-step RT-PCR kit with SYBR green was used with a thermal cycler (iCycler, Bio-Rad, USA) for RT-qPCR. Primers were used at final concentrations of 0.2 μM, and 10 ng of cDNA was used as a template in each 20-μL reaction. The threshold cycles for each sample were calculated based on fluorescence data using proprietary software (Bio-Rad, USA).

### Enzyme activity

To measure enzyme activity, cells were harvested at the onset of anaerobic fermentation (15 h) via centrifugation at 12,000 × g and 4 °C. The cells were resuspended in 100 mM Tris-HCl (pH 7.4) and disrupted on ice for 15 min (with a working period of 5 s and a 7-s interval constituting each cycle) at a power output of 200 W using an ultrasonic disruptor (J92-II, Xinzhi, Ningbo, China). Unbroken cells were removed by centrifugation at 10,000 × g for 20 min, and the supernatant was further centrifuged at 10,000 × g for 10 min. The resultant supernatant was used in the enzyme activity assays.

All the enzyme activity assay protocols have been described in previous studies and were optimized for the conditions and media used in this study. Phosphofructokinase (PFK)^19^, phosphoenolpyruvate carboxykinase (PCK), phosphoenolpyruvate carboxylase (PPC), isocitrate lyase (ICL)^20^ and malate synthetase (MS)^21^, malate dehydrogenase (MDH)^22^, fumarate hydratase (FH)^23^, citrate synthetase (CS)^24^, isocitrate dehydrogenase (IDH)^25^, and succinyl coenzyme A synthetase (SCS)^26^ were measured. The rate of DNTB increase was measured at 412 nm, and the corresponding extinction coefficient used was 13.6 cm^−1^mM^−1^. The wavelength and millimolar extinction coefficient used to determine NAD^+^, NADH, NADP^+^, and NADPH were 340 nm and 6.22 cm^−1^mM^−1^, respectively. Fumarate formation was recorded at 240 nm using an extinction coefficient of 2.53 cm^−1^mM^−1^. ATP and ADP were determined by HPLC at 254 nm^27^. One unit (U) of enzyme activity represents the amount of enzyme that catalyzes the conversion of 1 μmol of substrate per min into specific products. The total protein concentration in the crude cell extracts was measured using a BCA protein assay kit (Beyotime, China). Enzyme assays were performed in triplicate, and if the discrepancy between the results was greater than 10%, another pair of assays was performed.

### Expression and detection of Cra protein

To aid in the purification of Cra, a His-tag encoding fragment was included in the construct. After cloning the PCR product into pET28a, *E. coli* BL21 cells were transformed with the resultant pET-*cra* construct; the cells were then grown in LB medium at 37 °C to OD_600_ = 1.0. Gene overexpression was induced by adding 1 μM isopropyl-β-D- thiogalactopyranoside (IPTG) (Biosharp, Seoul, Korea), and the cells were cultured overnight. The cells were then centrifuged at 4,600 × g for 15 min, and the pellets were resuspended in phosphate-buffered saline (PBS) (pH 7.4), after which the cells were disrupted on ice using an ultrasonic disruptor (J92-II, Xinzhi, Ningbo, China). Unbroken cells were removed by centrifugation at 10,000 × g for 30 min. Finally, the proteins were resuspended in 100 mM Tris buffer (pH 6.8) (ANGUS, USA) containing 10% β-mercaptoethanol (AMRESCO, USA), and the suspension was stored at −80 °C. For purification, the native His_6_-Cra protein was isolated from the cell lysate supernatant using Ni metal affinity resin (Clontech, USA) according to the manufacturer’s instructions. The purified protein was stored in elution buffer at 4 °C until use, as indicated in each case.

### Isothermal titration microcalorimetry (ITC)

ITC experiments were performed using a TAM III instruments (Q Series Thermal Analysis) at 25 °C. Cra was thoroughly desalted using PD-10 desalting columns (GE, USA). The protein concentration of the desalted solution was determined using a BCA protein assay kit. Each ITC titration involved 10-μL injections of 50 mM FBP into a 15 μM protein solution that was placed into the 1.4-mL chamber of the apparatus. Mean enthalpies measured after the injection of FBP into the buffer were subtracted from the raw titration data prior to data fitting using the one binding site model of the MicroCal version of ORIGIN software. Δ*H* (reaction enthalpy), *K*_A_ (binding constant), and n (reaction stoichiometry) were determined based on the fitted curves. The change in free energy (Δ*G*) and in entropy (Δ*S*) were calculated using the equation: Δ*G* = −*RT*ln*K*_A_ = Δ*H* − *T*Δ*S*, where *R* is the universal molar gas constant and *T* is the absolute temperature.

### Electrophoretic Mobility Shift Assay (EMSA)

EMSA was performed as previously described^28^. The 18-bp DNA fragment used for EMSA was prepared by heating a 50 nM mixture of complementary oligonucleotides containing the presumed Cra binding site of the *aceBAK* region in 1 mL of TE buffer at 95 °C for 10 min. The annealed product was maintained on ice until use. Cra was incubated with the DNA fragment in the presence of 1 mM FBP (Sigma, 98% purity). Reaction mixtures were incubated for 15 min at 30 °C and electrophoresed in a nondenaturing 10% (w/v) polyacrylamide gel. Finally, the gels were stained with ethidium bromide and visualized using a gel imaging system (Tanon-1600, China).

## Results and Discussion

### Isolation of high-yield succinate mutants

To isolate high-yield succinic acid mutants, error-prone PCR was performed to construct *cra* mutant libraries. More than 10,000 clones were isolated and cultured in 96-well plates, and the results were detected using HPLC. 16 strains with superior succinate concentrations were selected for further testing in anaerobic bottles. Among the 16 strains, 6 high-yield mutant strains were selected and sequenced. The average succinic acid concentration produced by the 6 mutants was 35.6 g/L (data not shown) representing a 20% higher concentration than that obtained using the parental AFP111strain. The 6 mutants (R57K, G59N + T76Y, G59Q, A58T + R149I, A58G + S75H, and R60Q + D148I) were designated Tang1535, Tang1536, Tang1537, Tang1538, Tang1539 and Tang1540, respectively (Table 1).

**Figure 1:**
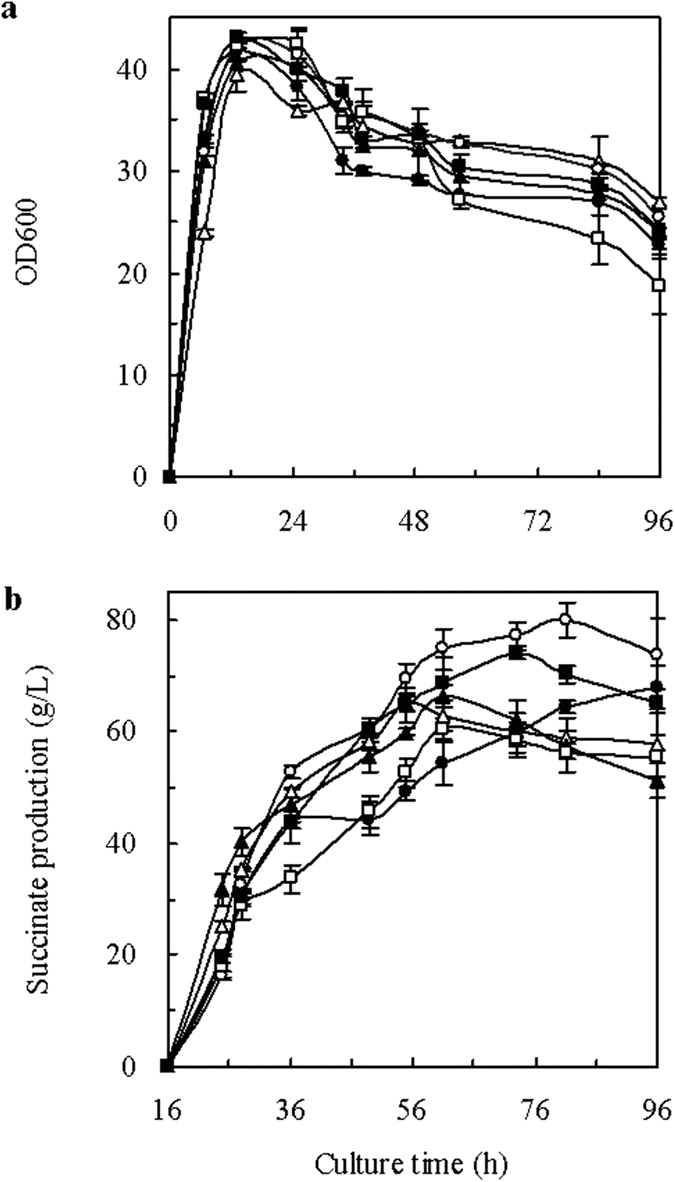
Effect of expression of the global transcription factor Cra on cell growth (**a**) and succinate production (**b**). *E. coli* strains: Tang1505 (pTrc99A) (open triangle, Δ); Tang1534 (Cra) (black triangle, ▲); Tang1541 (open circle, ○); Tang1542 (black circle, ●); Tang1543 (open square, □); and Tang1544 (black square, ■). The standard deviations were calculated from three independent experiments.

To further improve succinic acid biosynthesis, the mutations were introduced together into AFP111 to form 4 integrated mutant strains, which were designated Tang1541 to Tang1544 (Table 1). To test the fermentative production of succinate by these 4 integrated mutants, dual-phase fed-batch fermentations were conducted in a 7.5-L bioreactor. The aerobically grown cells were transferred to anaerobic conditions after cultivation for 15 h. The biomasses obtained for the 4 integrated mutants were all greater than those of the control strains (Tang1505 and Tang1534) (Fig. 1a). This result suggests that the *cra* mutations slightly promoted cell growth. The time profile of glucose consumption by the mutants was similar to that of Tang1505 (pTrc99A), illustrating that the mutations did not affect sugar consumption significantly (data not shown). The plasmids with the combined mutation showed improved effect on the exhibited increased succinate biosynthesis (Fig. 1b). The highest succinate concentrations (79.8 ± 3.1 and 74.4 ± 1.3 g/L) were obtained using Tang1541 (R57K + A58G + G59Q + R60Q + S75H + T76Y + D148I + R149I) and Tang1544 (R57K + A58T + G59N + R60Q + S75H + T76Y + D148I + R149I), respectively, representing 22.1% and 14.5% increases, respectively, compared to the concentration obtained using Tang1534 (Cra) (65.0 ± 1.5 g/L). The greatest succinate yield (1.23 mol/mol glucose) was obtained using Tang1544. The C4 metabolism ratios (C4 products per sum of C1 + C2 + C3 + C4) of Tang1541 and Tang1505 were 84.0% and 75.7%, respectively (Table 3). These results indicate that recombinant strains carrying Cra mutations effectively shifted their metabolic flux to succinate production.

### Cra mutation affects the expression and activity of genes and enzymes involved in succinate biosynthesis

**Figure 2:**
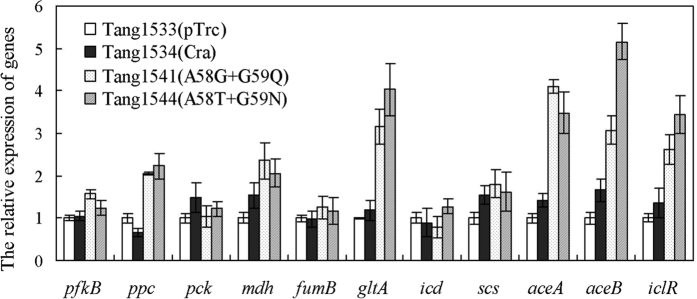
Effect of Cra mutation on the relative expression of genes involved in succinate metabolism. Three replicates were performed, and the error bars represent standard deviations.

To examine the gene expression profiles of the mutants, the mRNA transcripts from eleven genes were analyzed using semi-quantitative RT-qPCR (Fig. 2). The 11 genes, including *pfkB* (encoding phosphofructokinase), *ppc* (encoding phosphoenolpyruvate carboxylase), *pck* (encoding phosphoenolpyruvate carboxykinase), *mdh* (encoding malate dehydrogenase), *fumB* (encoding fumarate hydratase), *gltA* (encoding citrate synthetase), *icd* (encoding isocitrate dehydrogenase), *scs* (encoding succinyl coenzyme A synthetase), *aceA* (encoding isocitrate lyase), *aceB* (encoding malate synthetase), and *iclR* (encoding IclR transcriptional repressor), were selected based on their association with succinate biosynthesis.

The transcription of genes involved in (*aceA* and *aceB*) or related to (*iclR*) the glyoxylate pathway was up-regulated in both Tang1541 and Tang1544. Cra acts as an activator of genes that are involved in the glyoxylate shunt^15^. After mutation, this activation was enhanced. These obvious positive transcriptional influences might be useful for the biosynthesis of succinate. The expression of genes involved in the PEP carboxylation (*ppc*), reductive (*mdh*), and oxidative branches (*gltA*) of the TCA cycle was up-regulated in Tang1541 and Tang1544. This might have occurred due to the direct activation of *mdh* and *gltA* by the global transcription factor CRP (cyclic AMP receptor protein)^29^, and the gene encoding CRP might be activated by Cra^30^. Previous studies have demonstrated that *ppc* is repressed by Cra^31^. However, the Cra mutant exhibited the opposite regulatory effect, possibly due to structural and functional differences between Cra and the mutants. The expression of genes involved in the oxidative branches of the TCA cycle (*gltA*) was up-regulated in Tang1541 and Tang1544.

**Figure 3:**
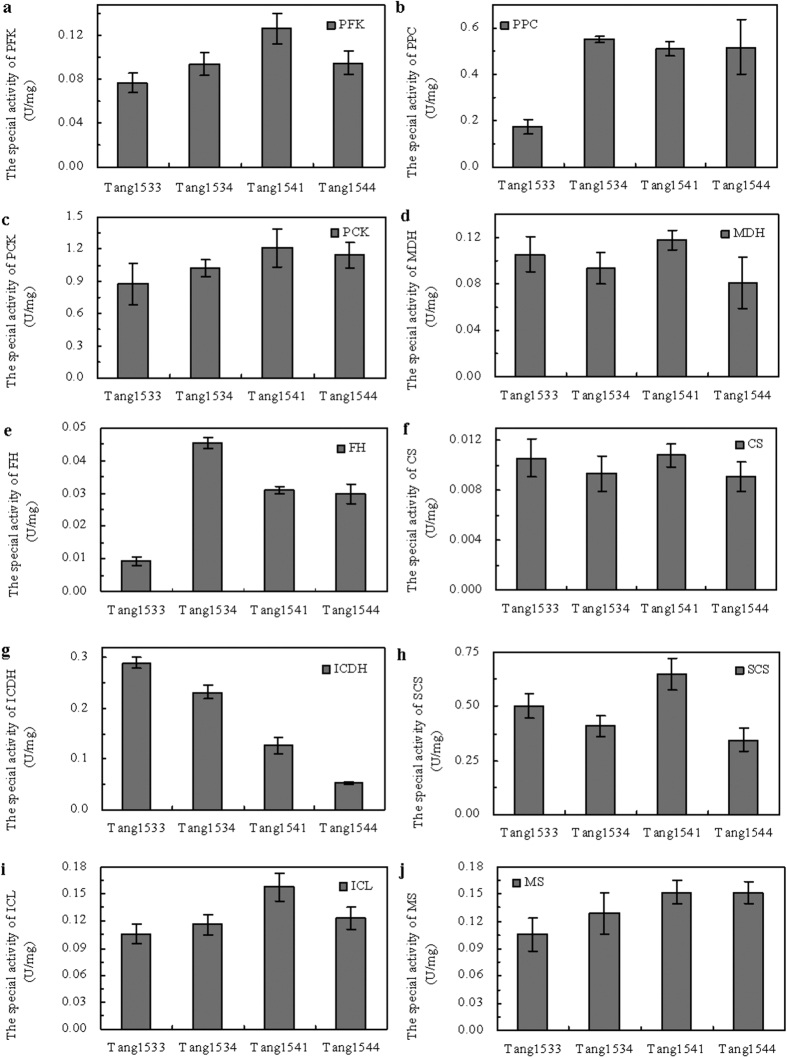
Effect of Cra mutation on the activity of enzymes involved in succinate metabolism. Central metabolism of *E. coli*. PFK, phosphofructokinase (**a**); PPC, PEP-carboxylase (**b**); PCK, PEP-carboxykinase (**c**); MDH, malate dehydrogenase (**d**); FH, fumarate hydratase (**e**); CS, citrate synthase (**f**); ICDH, isocitrate dehydrogenase (**g**); SCS, succinyl-CoA synthetase (**h**); ICL, isocitrate lyase (**i**); and MS, malate synthetase (**j**). Three replicates were performed, and the error bars represent standard deviations.

Figure 3 shows the activities of ten enzymes (involved in pathways that affect succinate production) that were measured in Tang1541, Tang1544 and two control strains during the fermentation period. The results reveal the main intermediary metabolism responses after transcription factor mutation. The following enzymes were examined: phosphofructokinase (PFK), phosphoenolpyruvate carboxykinase (PCK), phosphoenolpyruvate carboxylase (PPC), malate dehydrogenase (MDH), fumarate hydratase (FH), citrate synthetase (CS), isocitrate dehydrogenase (ICDH), succinyl coenzyme A synthetase (SCS), isocitrate lyase (ICL), and malate synthetase (MS).

PFK activity was highest in Tang1541 and was lowest in Tang1505 (pTrc99A) (Fig. 3a). This result indicates that PFK was activated by the mutation of Cra although previous studies showed that Cra represses the glycolytic pathway^14^. Greater PFK activities would be expected to drive a more active glycolysis pathway. The active glycolysis pathway might increase the concentration of phosphoenolpyruvate (PEP), which might improve the activity of carboxylation enzymes as substrates. As shown in Fig. 3b, PPC activity was highest in Tang1534 (Cra). This suggests that PPC was activated by the overexpression of Cra but not by the mutation of Cra. However, *ppc* expression was up regulated in Tang1541 and Tang1544. As shown in Fig. 3c, PCK activity was higher in Tang1541 and Tang1544 than in Tang1505 (pTrc99A) or Tang1534 (Cra). This higher PCK and PPC activity enhanced CO_2_ fixation (Table 3), which is considered the key step in succinic acid synthesis^32^. The mutation of Cra did not significantly alter MDH activity (Fig. 3d). FH was activated by the overexpression of Cra but not by the mutation of Cra (Fig. 3e); however, the FH activity observed in the Cra mutant (Tang1541 and Tang1544) was still greater than that observed in cells with empty vectors (Tang1505).

CS activity did not significantly differ between the Cra mutant and the control strains (Fig. 3f). ICDH, which catalyzes the conversion of isocitrate to α-ketoglutarate, is regulated by post-transcriptional modification involving phosphorylation. This phosphorylation is catalyzed by the protein AceK, which is encoded by the *aceBAK* operon. ICDH activity was minimal in the Tang1541 and Tang1544 mutants (Fig. 3g), whereas SCS activity was highest in Tang1541 (Fig. 3h).

Glyoxylate enzyme (ICL and MS) activity was highest in the Cra mutant (Tang1541 and Tang1544) (Fig. 3i,j). Previous research has shown that the glyoxylate pathway is an important pathway for succinate production^6^,^33^. The obvious enhancement of the gene expression and activity of glyoxylate pathway enzymes might be useful for succinate biosynthesis.

In summary, the enzymes involved in phosphoenolpyruvate (PEP) carboxylation, reductive branches of the TCA cycle and the glyoxylate pathway were activated by the mutation of Cra, either directly or indirectly. This might explain the significant increase in succinate production.

### Amino acid mutations in Cra

**Figure 4:**
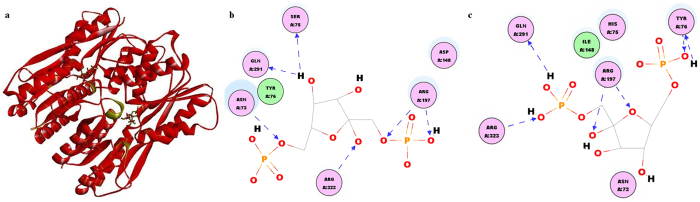
Amino acid mutations in Cra. The 3D structure of the mutated transcription factor (**a**). Details of the interaction between Cra and FBP (**b**). Details of the interaction between the Cra mutant and FBP (**c**). The structural stereoview was prepared using Discovery Studio with the native Cra structure as a template (PDB: 2IKS).

The Cra protein of *E. coli* occurs predominantly in a tetramer form and comprises two functional domains: an N-terminal DNA-binding domain with an H-T-H (helix-turn-helix) motif and a C-terminal inducer-binding and subunit-subunit contact domain^31^. A structural stereoview was prepared using Discovery Studio 3.0 with the native Cra structure as the template (PDB: 2IKS). The structures of Cra and the Cra mutant are shown in Fig. 4a. The amino acid mutations are located in the binding cavity of the effector. This finding suggests that the mutation site might affect the target gene expression by affecting the affinity of Cra for the effector FBP^34^. As shown in Fig. 4b,c, 6 hydrogen bonds are present in the model of both the wild-type Cra and the Cra mutant. The mutations in Cra resulted in 2 additional hydrogen bonds involving the amino acid of Tyr-76; however, the hydrogen bonds observed in wild-type Cra at Asn-73 and Ser-75 were lost. From the software simulation results, the free energy change in the Cra-FBP binding energy was 1.98 kJ/mol after mutation (data not shown). This indicates that the mutation enhanced the binding of Cra to FBP and suggests that the improved succinate production by the Cra mutant was probably associated with the improved affinity of Cra for FBP.

### ITC assays with Cra and FBP

**Figure 5:**
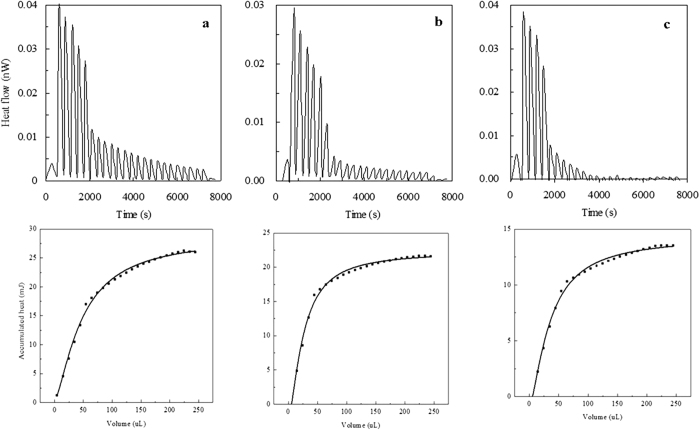
ITC assays with Cra and FBP. Isothermal titration calorimetry measurements to determine the ligand profile of Cra at 25 °C. The upper panels show raw titration data, and the lower panels show integrated and dilution-corrected peak area plots of the titration data. For the titration, Cra (10–15 μM) was introduced into the sample cells, and the ligand concentration in the buffer was 50 mM. Titration of FBP with the Cra mutants Tang1534 (**a**), Tang1541 (**b**) and Tang1544 (**c**). Thermodynamic values calculated for the Cra-FBP interaction (Tang1534) were as follows: *K*_*d*_ = 1,400 ± 150 nM, Δ*H* = −16 ± 2 MJ/mol, *T*Δ*S* = −14.664 MJ/mol, and Δ*G* = −18.038 kJ/mol. Thermodynamic values calculated for the Cra-FBP interaction (Tang1541) were as follows: *K*_*d*_ = 130 ± 37 nM, Δ*H* = −9.4 ± 1.7 MJ/mol, *T*Δ*S* = 8.585 MJ/mol, and Δ*G* = −12.116 kJ/mol. Thermodynamic values calculated for the Cra-FBP interaction (Tang1544) were as follows: *K*_*d*_ = 95 ± 24 nM, Δ*H* = −6.2 ± 1.0 MJ/mol, *T*Δ*S* = 5.632 MJ/mol, and Δ*G* = −11.301 kJ/mol.

To verify whether the affinity of the Cra mutant for FBP was enhanced, the interaction parameters of the Cra protein with FBP were examined using ITC. As shown in the upper panel of Fig. 5a, the peaks are positive, indicating that the binding of wild-type Cra to FBP was exothermic (Δ*H* = −16 ± 2 MJ/mol, *T*Δ*S* = −14.664 MJ/mol, Δ*G* = −18.038 kJ/mol). Subsequent calculations allowed us to determine a Cra-FBP dissociation constant (*K*_*d*_) of 1,400 ± 150 nM, reflecting an interaction between FBP and wild-type Cra. The results shown in Fig. 5b indicate that the binding of FBP to the Cra mutant (Tang1541) was both enthalpy-driven (Δ*H* = −9.4 ± 1.7 MJ/mol) and entropy-driven (*T*Δ*S* = 8.585 MJ/mol) and produced a total free energy change of Δ*G* = −12.116 kJ/mol. Favorable (negative) enthalpy changes can be attributed to hydrogen bonding or to van der Waals forces between FBP and amino acids at a specific binding site and mode in the Cra mutant. Further calculations revealed a high protein-effector affinity (*K*_*d*_ = 130 ± 37 nM). The interaction results obtained for the Tang1544 Cra mutant with FBP were similar to those obtained for Tang1541 (Fig. 5c). This interaction was both enthalpy-driven (Δ*H* = −6.2 ± 1.0 MJ/mol) and entropy-driven (*T*Δ*S* = 5.632 MJ/mol), producing a total free energy change of Δ*G* = −11.301 kJ/mol. Moreover, the protein-effector affinity was high (*K*_*d*_ = 95 ± 24 nM). FBP had a stronger binding affinity for the Cra mutant, possibly affecting the transcriptional regulation capacity of Cra. This suggests that the improvement in succinate production observed for the Cra mutant was associated with the improved affinity of Cra for FBP.

### Nonradioactive EMSA of Cra and its binding site in the *aceBAK*

**Figure 6:**
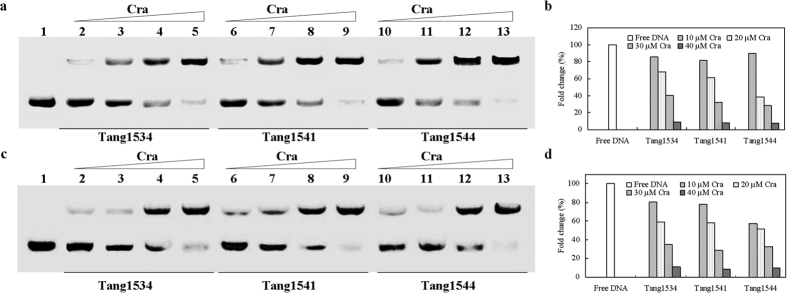
Nonradioactive EMSA of Cra and its binding site in *aceBAK*. EMSA experiment using 0.75 μM of the Cra site-containing DNA fragment and increasing concentrations of wild type Cra or the Cra mutant proteins in the presence of FBP (**a**) and the gray value of free DNA determined using Quantity one (**b**) and in the absence of FBP (**c**) and the gray value of free DNA determined using Quantity one (**d**). Lane 1, no protein; lane 2–5, 1, 2, 3, 4 μM Cra; lanes 6–9, 1, 2, 3, 4 μM Cra mutant (Tang1541); lanes 10–13, 1, 2, 3, 4 μM Cra mutant (Tang1544), respectively.

Cra binds to target promoter regions to regulate gene expression. The enhanced affinity of the Cra mutant for FBP might regulate the Cra-regulated gene expression. To examine whether *aceA* and *aceB* are activated by the Cra mutant, an 18-bp double-stranded DNA fragment containing the candidate target of the *aceBAK* regulatory region was synthesized for use in a mobility shift assay, as shown in Fig. 6. DNA samples were incubated with pure Cra protein and subjected to EMSA experiments. When pure Cra was used, the band corresponding to the complex disappeared, demonstrating that the binding to the protein was specific. As shown in Fig. 6a,c (lanes 2–13), the bands representing the formation of a specific complex between the DNA and the Cra protein. Gray scale analysis are shown in Fig. 6b,d; the amount of free DNA decreased with Cra concentration. With the addition of FBP, the binding of wild type Cra with DNA was weaker than that obtained in the absence of FBP. This may be due to the elimination of regulation effects of Cra on transcription caused by FBP. When FBP was absent, the abundance of free DNA from Tang1544 was less than that of Tang1534 (Fig. 6d), indicating that the Cra mutant bound DNA more strongly, which would enhance the expression of genes involved in glyoxylate pathway. Interestingly, when FBP was present, the abundance of free DNA from Tang1544 was further decreased. Although the affinity of the Cra mutant for FBP was enhanced, no significant negative effect on the expression of the genes was observed. It is anormal in comparison with the reported phenomena. According to Bledig, the regulation of *pykF* expression was counteracted when effector was added^35^. The result suggested the regulatory effect of the Cra mutant on *aceBAK* was altered. Some of the negative effect of FBP on Cra might have been counteracted through the enhanced binding affinity of the Cra mutant for FBP or the change of Cra structure. Thus the expression of genes associated with succinate biosynthesis were enhanced, and eventually contributing to succinate biosynthesis.

## Conclusions

To improve succinate production of *E. coli*, random mutagenesis libraries were generated using error-prone PCR of Cra. The best mutant strain, Tang1541, was observed after several rounds of screening. Genes involved in PEP carboxylation and the glyoxylate pathway were activated by the Cra mutant, thereby enhancing succinate production. The results of an EMSA confirmed that the Cra mutant bound to *aceBAK*, thereby activating the glyoxylate pathway and further improving succinate biosynthesis. Some of the negative effect of FBP on Cra might have been counteracted through the enhanced binding affinity of the Cra mutant for FBP or the change of Cra structure.

## Additional Information

**How to cite this article**: Zhu, L.-W. *et al*. Enhancing succinic acid biosynthesis in *Escherichia coli* by engineering its global transcription factor, catabolite repressor/activator (Cra). *Sci. Rep*. **6**, 36526; doi: 10.1038/srep36526 (2016).

**Publisher’s note:** Springer Nature remains neutral with regard to jurisdictional claims in published maps and institutional affiliations.

## Figures and Tables

**Table 1:** Strains and plasmids used in this study.

Strains	Relevant characteristics	Sources or reference
AFP111	F + λ- *rpo*S396(Am) rph-1 Δ*pflAB*::Cam *ldhA*::Kan *ptsG*	17
DH5α	F-φ80 *lacZ*ΔM15 Δ(*lacZYA*-*argF*)U169 *endA*1 *recA*1 *hsdR*17(r_k_-, m_k_-) *supE*44 λ-*thi*-1 *gyrA*96 *relA*1 *phoA*	TransGen Biotech
BL21	F- *ompT*, *hsdS*_B_ (r_B_- m_B_-), *gal*, *dcm*	TransGen Biotech
Tang1505	AFP111/pTrc99A	This study
Tang1534	AFP111/pTrc-*cra*	This study
Tang1535	AFP111/pTrc-*cra* with mutation at R57K	This study
Tang1536	AFP111/pTrc-*cra* with mutation at G59N + T76Y	This study
Tang1537	AFP111/pTrc-*cra* with mutation at G59Q	This study
Tang1538	AFP111/pTrc-*cra* with mutation at A58T + R149I	This study
Tang1539	AFP111/pTrc-*cra* with mutation at A58G + S75H	This study
Tang1540	AFP111/pTrc-*cra* with mutation at R60Q + D148I	This study
Tang1541	AFP111/pTrc-*cra* with mutation at R57K + A58G + G59Q + R60Q + S75H + T76Y + D148I + R149I	This study
Tang1542	AFP111/pTrc-*cra* with mutation at R57K + A58T + G59Q + R60Q + S75H + T76Y + D148I + R149I	This study
Tang1543	AFP111/pTrc-*cra* with mutation at R57K + A58G + G59N + R60Q + S75H + T76Y + D148I + R149I	This study
Tang1544	AFP111/pTrc-*cra* with mutation at R57K + A58T + G59N + R60Q + S75H + T76Y + D148I + R149I	This study
Plasmids		
pTrc99A	*Ap*^*R*^, pBR322 ori, *trc* promoter, *lacI*^*q*^	Invitrogen
pET28a	*Kan*^*R*^, pBR322 ori, *lacI*^*q*^, T7 promoter	Novagene
pTrc-*Cra*	pTrc99A carrying *cra* gene	This study
pET-*Cra*	pET28a carrying *cra* gene	This study
pET-*CGQ*	pET28a carrying *cra* gene with mutation at R57K + A58G + G59Q + R60Q + S75H + T76Y + D148I + R149I	This study
pET-*CTN*	pET28a carrying *cra* gene with mutation at R57K + A58T + G59N + R60Q + S75H + T76Y + D148I + R149I	This study

**Table 2:** Primers used in this study^a^.

Primer (5′-3′)	Relevant characteristics
*cra*-F	AGCT***GAATTC***GTGAAACTGGATGAAATCGCTCG
*cra*-R	AATT***GGATCC***TTAGCTACGGCTGAGCACGCCGC
*Cra*-SacI-F	GATC***GAGCTC***GTGAAACTGGATGAAATCGCTCGG
*Cra*-HindIII-His-R	ACAG***AAGCTT***TTA**GTGATGGTGATGGTGATG**GCTACGGCTGAGCA
*gltA*-F(RT)	TTACCCGTCTGTTCCATGCT
*gltA*-R(RT)	CACGGTGACGAGGATTGTT
*iclR*-F(RT)	GGTCAATATGGCGGTGCTT
*iclR*-R(RT)	CTTCGCTCAGTTGGGCTAAA
*icd*-F(RT)	AGGTTTATGGTCAGGACG
*icd*-R(RT)	GCAGGCAGATGTAGAGAT
*aceA*-F(RT)	GTCGGATATGGGCTACAA
*aceA*-R(RT)	TTCCTGCTGGTGAGATAC
*aceB*-F(RT)	GGCAGTGACGATGGATAA
*aceB*-R(RT)	GTGACCGTTATTGGCTTC
*pfkB*-F(RT)	CAGCACTGGCAATTGGTAACA
*pfkB*-R(RT)	TTTGGCCTTGCCGCTATT
*Succinyl-CoA*-F(RT)	AAATGTCAGGTTCACGCTGGTG
*Succinyl-CoA*-R(RT)	CGAGATACAGCTCTTTAGCG
*fumB*-F(RT)	CGGTAGATGGCGATGAGTAC
*fumB*-R(RT)	*ACCGAGGGTACGCATTTT*
*mdh*-F(RT)	CCCGAAAGCGTGCATTGGTA
*mdh*-R(RT)	TGGCTGTTTGCCTTTCAG
*pck*-F(RT)	TTTGCAGGCGCTGACCGCAATTAC
*pck* -R(RT)	CAGCTGGATCAGCTTTGAAGTTCG
*ppc* -F(RT)	AACCATTGCCAGTGGGCATTGAC
*ppc* -R(RT)	CCGGATGGTGTGACGGACAATTTC
16S rRNA-F	GCTAATACCGCATAACGTCGCAAG
16S rRNA-R	GGACCGTGTCTCAGTTCCAGTGTG
*aceBAK*-F	TCGTTAAGCGATTCAGCA
*aceBAK*-R	TGCTGAATCGCTTAACGA

^a^Italic and bold bases encode restriction site and underlined bases encode 6 * His tag.

**Table 3:** Effect of Cra mutation on the production of fermentation products.

	Tang1505	Tang1534	Tang1541	Tang1542	Tang1543	Tang1544
Succinate productivity (g/L h)	1.18	1.09	0.99	0.71	0.99	1.01
Succinate yield (mol/mol glucose)	1.09	1.02	1.21	0.71	0.81	1.23
Lactate production (g/L)	5.6 ± 0.3	4.4 ± 0.5	4.8 ± 0.4	2.5 ± 0.6	4.9 ± 0.3	3.3 ± 0.1
Lactate yield (mol/mol glucose)	0.11	0.08	0.09	0.03	0.08	0.05
Acetate production (g/L)	5.1 ± 0.5	4.8 ± 0.6	3.6 ± 0.3	6.5 ± 0.4	9.2 ± 0.6	6.1 ± 0.4
Acetate yield (mol/mol glucose)	0.15	0.13	0.10	0.13	0.23	0.14
Formate production (g/L)	2.3 ± 0.1	1.5 ± 0.2	1.0 ± 0.1	2.2 ± 0.1	1.5 ± 0.1	1.7 ± 0.1
Formate yield (mol/mol glucose)	0.09	0.05	0.04	0.06	0.05	0.05
C4 metabolism ration (%)^a^	75.7	79.7	84.0	76.3	69.2	83.7

^a^C4 metabolism ratio represents the C4 molar yield per sum of C1 + C2 + C3 + C4 molar yield.
